# Supplementary material for: Identification of AtHsp90.6 involved in early embryogenesis and its structure prediction by molecular dynamics simulations
Source: R Soc Open Sci. 2019 May 1;6(5):190219. doi: 10.1098/rsos.190219 (PMC6550000; doi:10.1098/rsos.190219)
Supplement: Supplemental figures, tables and data [file rsos190219supp1.doc]

Identification of AtHsp90.6 involved in early embryogenesis and its structure prediction by molecular dynamics simulations

**An Luo^1^, Xinbo Li^2,3^, Xuecheng Zhang^2^, Huadong Zhan^4^, Hewei Du^1^,Yubo Zhang^5*^, and Xiongbo Peng^2*^**

**
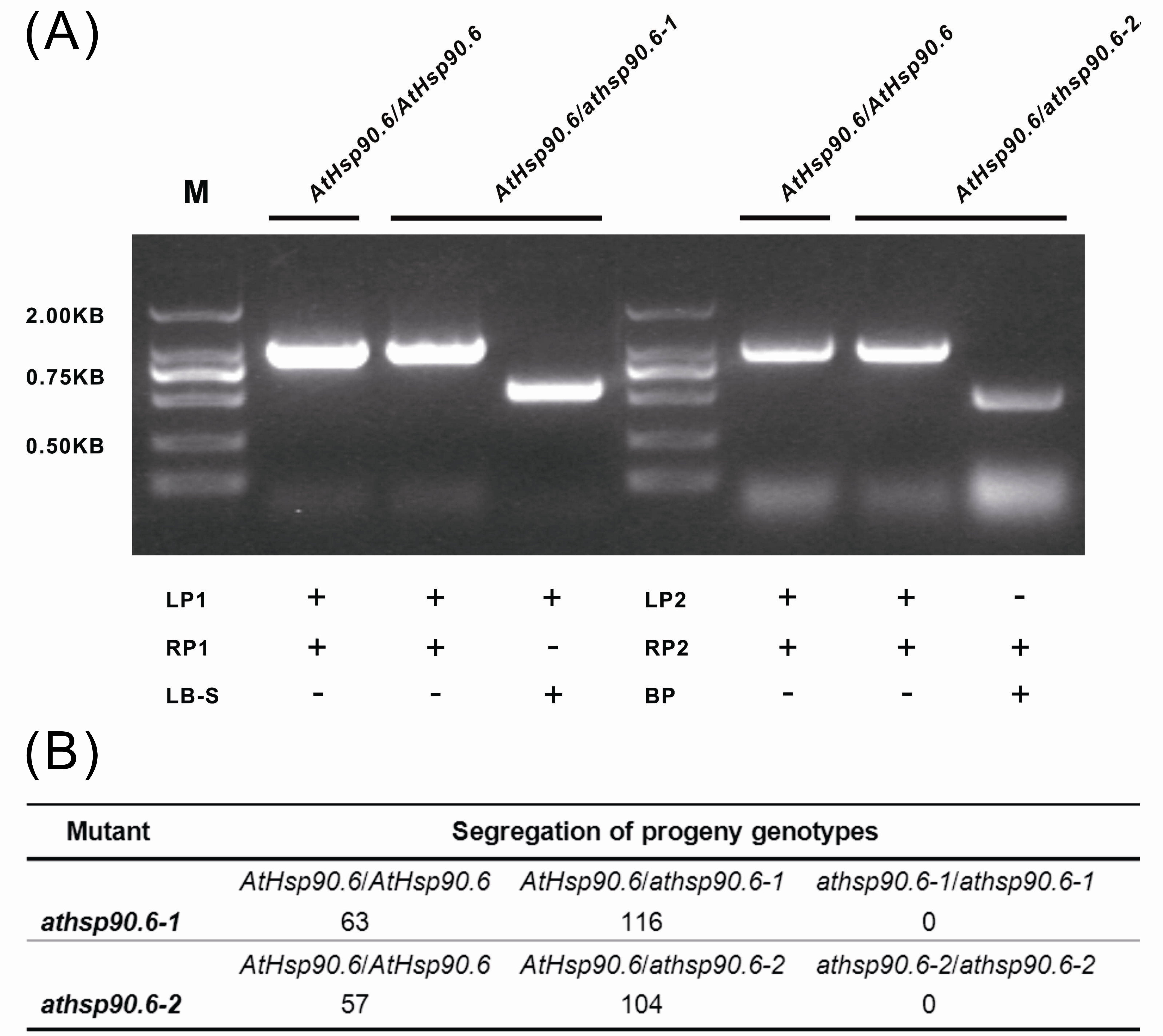
**

**Figure S1. Genotype identification and progeny genotypes of *athsp90.6* mutant** (A) Genotypes of *AtHsp90.6*/*AtHsp90.6*, *AtHsp90.6*/*athsp90.6-1*, *and AtHsp90.6*/*athsp90.6-2* could be confirmed by PCR analysis. M, marker; Primer LP1 and RP1, LP2 and RP2 were used for identification of *AtHsp90.6* gene respectively; Primer LP1 and LB-S, RP2 and BP were used for identification of T-DNA insertion respectively.(B) Segregation of progeny genotypes of *athsp90.6-1* and *athsp90.6-2* mutants.


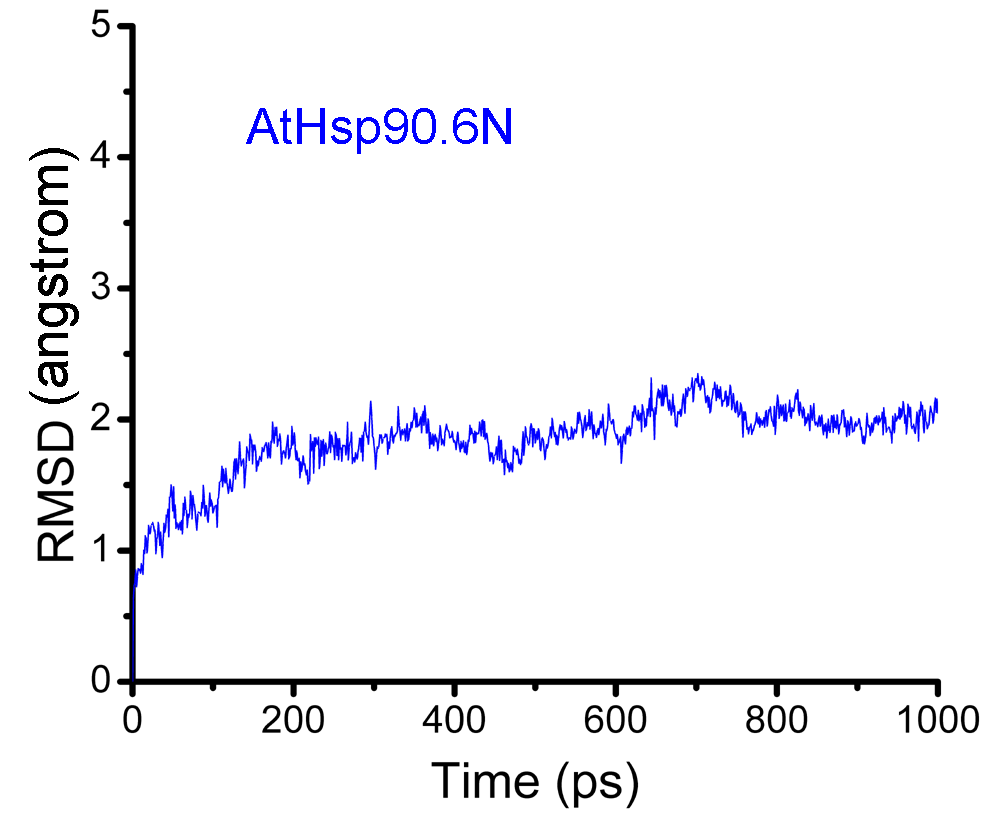


**Figure S2. Time evolution of the RMSD of AtHsp90.6N along 1000 ps simulations.**


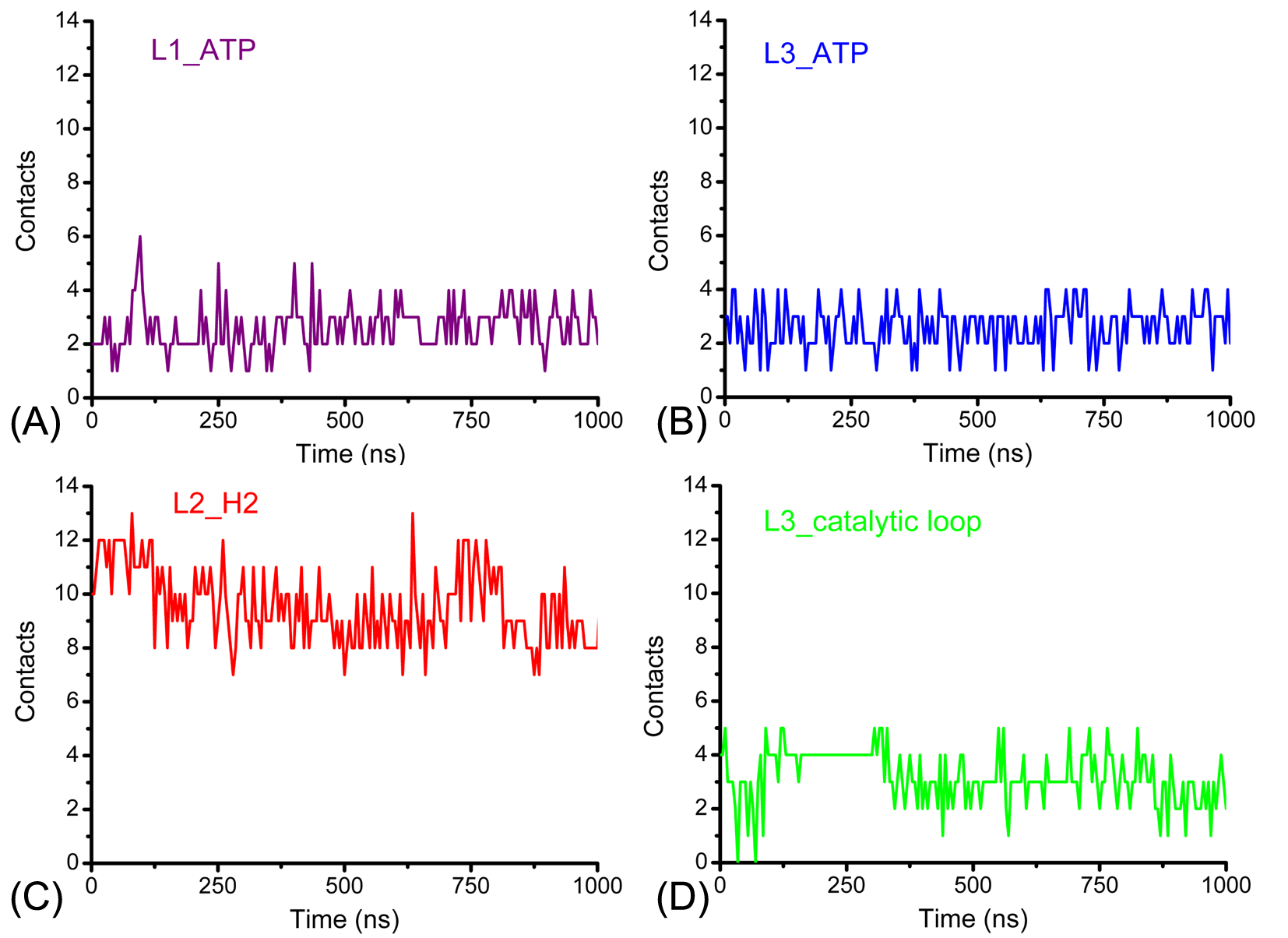


**Figure S3. The number of contacts in L1-ATP (A), L3-ATP (B), L2-H2(C) and L3-catalytic loop (D) along 1000 ns simulations.**

**Table S1. Complementation of the seed abortion phenotype in complementation lines (*athsp90.6-1*/*athsp90.6-1*, g-AT3g07770/g-AT3g07770)**

| Complementation line | Fertile  seed | Seed  set ratio(%) | | P value for *athsp90.6-1*  Seed set ratio (646:877) | n |
| --- | --- | --- | --- | --- | --- |
| 1 | 413 | 98.33 | 6.097×10^-119**^ | | 420 |
| 2 | 398 | 98.27 | 1.628×10^-114**^ | | 405 |
| 3 | 440 | 96.70 | 2.079×10^-121**^ | | 455 |
| 4 | 429 | 97.28 | 3.349×10^-120**^ | | 441 |
| 5 | 407 | 98.78 | 1.400×10^-118**^ | | 412 |

**Validation by χ2 test. **Significant difference with the probability value P<0.01.

**Table S2. Summary of simulations**

| Protein | Ligand | Time [ns] | Force field | Tools |
| --- | --- | --- | --- | --- |
| AtHsp90.6N | ATP | 100 | FF99 | Gromacs |
| AtHsp90.6N | ADP | 100 | FF99 | Gromacs |
| AtHsp90.6N | apo | 100 | FF99 | Gromacs |
| AtHsp90.6N | ATP | 1 | FF99 | Amber 11 |
| HsHsp90N | ATP | 1 | FF99 | Amber 11 |
| AtHsp90.6N | ADP | 1 | FF99 | Amber 11 |
| DdHsp90N | ADP | 1 | FF99 | Amber 11 |
| AtHsp90.6FL | ATP | 1000 | MARTINI | Gromacs |

**The AMBER parameter for ADP and ATP**

R-ADENOSINE - with diphosphate linker

adp.db94

adp INT 1

CORRECT OMIT DU BEG

0.0

1 DUMM DU M 0 0 0 0.000 0.000 0.000 0.000

2 DUMM DU M 1 0 0 1.000 0.000 0.000 0.000

3 DUMM DU M 2 1 0 1.000 90.000 0.000 0.000

4 O1B O3 M 3 2 1 1.000 90.000 180.000 -0.9552

5 PB P M 4 3 2 1.434 90.000 180.000 1.3672

6 O2B O3 E 5 4 3 1.574 107.490 -79.441 -0.9552

7 O3B O3 E 5 4 3 1.518 118.007 165.990 -0.9552

8 O3A OS M 5 4 3 1.599 113.374 180.000 -0.6346

9 PA P M 6 5 4 1.646 130.619 36.624 1.4929

10 O1A O2 E 7 6 5 1.504 109.212 -90.234 -0.9474

11 O2A O2 E 7 6 5 1.526 108.570 40.323 -0.9474

12 O5* OS M 7 6 5 1.585 97.173 157.726 -0.6579

13 C5* CT M 8 7 6 1.445 122.292 -66.317 0.0558

14 H50 H1 E 9 8 7 1.059 109.484 27.531 0.0679

15 H51 H1 E 9 8 7 1.059 109.437 -92.456 0.0679

16 C4* CT M 9 8 7 1.477 113.286 147.538 0.1065

17 H40 H1 E 16 9 8 1.059 105.768 -179.178 0.1174

18 O4* OS S 16 13 9 1.482 106.235 -62.889 -0.3548

19 C1* CT B 18 16 13 1.391 107.688 128.804 0.0394

20 H10 H2 E 19 18 16 1.059 109.468 91.228 0.2007

21 N9 N* S 19 18 16 1.552 105.091 -143.300 -0.0251

22 C8 CK B 21 19 18 1.388 123.797 33.434 0.2006

23 H80 H5 E 22 21 19 1.078 131.047 -3.389 0.1553

24 N7 NB S 22 21 19 1.384 108.967 176.598 -0.6073

25 C5 CB S 24 22 21 1.395 103.021 0.702 0.0515

26 C6 CA B 25 24 22 1.412 128.288 178.252 0.7009

27 N6 N2 B 26 25 24 1.333 125.907 -3.791 -0.9019

28 H60 H E 27 26 25 1.010 120.010 2.829 0.4115

29 H61 H E 27 26 25 1.009 120.014 -177.167 0.4115

30 N1 NC S 26 25 24 1.343 113.319 178.851 -0.7615

31 C2 CQ B 30 26 25 1.340 123.433 -1.664 0.5875

32 H2 H5 E 31 30 26 1.077 120.022 -177.875 0.0473

33 N3 NC S 31 30 26 1.377 125.379 2.123 -0.6997

34 C4 CB E 33 31 30 1.300 108.242 -0.035 0.3053

35 C3* CT M 16 13 12 1.484 117.051 57.581 0.2022

36 H30 H1 E 35 16 13 1.059 117.939 24.820 0.0615

37 O3* OH S 35 16 13 1.407 108.509 149.908 -0.6541

38 H3* HO E 37 35 16 0.967 109.470 163.254 0.4376

39 C2* CT M 35 16 13 1.607 102.425 -96.715 0.0670

40 H20 H1 E 39 35 16 1.059 115.086 78.119 0.0972

41 O2* OH S 39 35 16 1.398 114.943 -153.294 -0.6139

42 H2* HO E 41 39 35 0.967 109.456 -41.679 0.4186

IMPROPER

C8 C4 N9 C1*

C6 H60 N6 H61

N7 N9 C8 H80

N1 N3 C2 H2

C5 N1 C6 N6

LOOP CLOSING EXPLICIT

C1* C2*

C4 C5

C4 N9

DONE

STOP

R-ADENOSINE - with triphosphate linker

atp.db94

atp INT 1

CORRECT OMIT DU BEG

0.0

1 DUMM DU M 0 0 0 1.000 90.000 180.000 0.000

2 DUMM DU M 1 0 0 0.967 90.000 180.000 0.000

3 DUMM DU M 2 1 0 0.967 109.443 180.000 0.000

4 O1G O3 M 3 2 1 1.086 160.811 148.238 -0.9526

5 PG P M 4 3 2 1.379 92.848 -159.681 1.2650

6 O2G O3 E 5 4 3 1.417 116.381 -173.072 -0.9526

7 O3G O3 E 5 4 3 1.443 104.744 40.954 -0.9526

8 O3B OS M 5 4 3 1.742 94.444 -63.733 -0.5322

9 PB P M 8 5 3 1.512 135.464 -125.176 1.3852

10 O1B O2 E 9 8 5 1.415 113.542 -28.935 -0.8894

11 O2B O2 E 9 8 5 1.617 103.881 -164.747 -0.8894

12 O3A OS M 9 8 5 1.734 96.237 83.499 -0.5689

13 PA P M 12 9 8 1.516 131.613 -127.461 1.2532

14 O1A O2 E 13 12 9 1.526 115.352 -92.892 -0.8799

15 O2A O2 E 13 12 9 1.410 109.213 53.806 -0.8799

16 O5* OS M 13 12 9 1.749 97.280 164.349 -0.5987

17 C5* CT M 16 13 12 1.427 117.355 64.264 0.0558

18 H50 H1 E 17 16 13 1.059 109.461 103.970 0.0679

19 H51 H1 E 17 16 13 1.059 109.491 -16.065 0.0679

20 C4* CT M 17 16 13 1.446 107.888 -136.043 0.1065

21 H40 H1 E 20 17 16 1.059 106.572 -167.965 0.1174

22 O4* OS S 20 17 16 1.577 105.136 -48.024 -0.3548

23 C1* CT B 22 20 17 1.562 99.310 148.198 0.0394

24 H10 H2 E 23 22 20 1.059 104.667 127.006 0.2007

25 N9 N* S 23 22 20 1.651 99.707 -129.444 -0.0251

26 C8 CK B 25 23 22 1.400 121.683 69.274 0.2006

27 H80 H5 E 26 25 23 1.077 128.168 2.532 0.1553

28 N7 NB S 26 25 23 1.327 111.806 -177.463 -0.6073

29 C5 CB S 28 26 25 1.365 104.681 2.256 0.0515

30 C6 CA B 29 28 26 1.448 131.894 -177.019 0.7009

31 N6 N2 B 30 29 28 1.312 123.747 -3.485 -0.9019

32 H60 H E 31 30 29 1.000 120.000 176.690 0.4115

33 H61 H E 31 30 29 1.000 120.000 -3.310 0.4115

34 N1 NC S 30 29 28 1.362 114.694 173.408 -0.7615

35 C2 CQ B 34 30 29 1.370 122.869 5.034 0.5875

36 H2 H5 E 35 34 30 1.078 119.995 174.203 0.0473

37 N3 NC S 35 34 30 1.309 125.548 -5.799 -0.6997

38 C4 CB E 37 35 34 1.340 112.610 1.051 0.3053

39 C3* CT M 20 17 16 1.539 120.245 66.848 0.2022

40 H30 H1 E 39 20 17 1.059 109.025 -37.638 0.0615

41 O3* OH S 39 20 17 1.390 114.084 86.172 -0.6541

42 H3' HO E 41 39 20 0.967 109.487 -2.526 0.4376

43 C2* CT M 39 20 17 1.606 106.034 -155.382 0.0670

44 H20 H1 E 43 39 20 1.059 123.901 158.202 0.0972

45 O2* OH S 43 39 20 1.368 107.234 -72.414 -0.6139

46 H2' HO E 45 43 39 0.967 109.442 -16.862 0.4186

IMPROPER

C8 C4 N9 C1*

C6 H60 N6 H61

N7 N9 C8 H80

N1 N3 C2 H2

C5 N1 C6 N6

LOOP CLOSING EXPLICIT

C1* C2*

C4 C5

C4 N9

DONE

STOP
